# Cancer‐associated fibroblasts in pancreatic cancer: new subtypes, new markers, new targets

**DOI:** 10.1002/path.5926

**Published:** 2022-06-07

**Authors:** Shinelle Menezes, Mohamed Hazem Okail, Siti Munira Abd Jalil, Hemant M Kocher, Angus J M Cameron

**Affiliations:** ^1^ Barts Cancer Institute, Queen Mary, University of London, John Vane Science Centre London UK; ^2^ Barts and the London HPB Centre, The Royal London Hospital Barts Health NHS Trust London UK

**Keywords:** cancer‐associated fibroblasts, immunotherapy, *in vivo* models, knock‐out models, myofibroblast, pancreatic cancer

## Abstract

Cancer‐associated fibroblasts (CAFs) have conflicting roles in the suppression and promotion of cancer. Current research focuses on targeting the undesirable properties of CAFs, while attempting to maintain tumour‐suppressive roles. CAFs have been widely associated with primary or secondary therapeutic resistance, and strategies to modify CAF function have therefore largely focussed on their combination with existing therapies. Despite significant progress in preclinical studies, clinical translation of CAF targeted therapies has achieved limited success. Here we will review our emerging understanding of heterogeneous CAF populations in tumour biology and use examples from pancreatic ductal adenocarcinoma to explore why successful clinical targeting of protumourigenic CAF functions remains elusive. Single‐cell technologies have allowed the identification of CAF subtypes with a differential impact on prognosis and response to therapy, but currently without clear consensus. Identification and pharmacological targeting of CAF subtypes associated with immunotherapy response offers new hope to expand clinical options for pancreatic cancer. Various CAF subtype markers may represent biomarkers for patient stratification, to obtain enhanced response with existing and emerging combinatorial therapeutic strategies. Thus, CAF subtyping is the next frontier in understanding and exploiting the tumour microenvironment for therapeutic benefit. © 2022 The Authors. *The Journal of Pathology* published by John Wiley & Sons Ltd on behalf of The Pathological Society of Great Britain and Ireland.

## Fibroblasts in cancer

All solid organs and, consequently all solid tumours, contain abundant populations of fibroblasts, making them key regulators of tumour biology. As in development, organogenesis, and wound healing, fibroblasts are critical for the establishment of tissue structure and integrity. Fibroblasts are the predominant source and regulators of the extracellular matrix (ECM). In turn, the ECM provides the structure necessary to support angiogenesis and the associated nutrient supply necessary to support organ function or tumour growth. In addition to providing tumour structure, numerous studies have implicated fibroblasts in the regulation of all aspects of tumour progression, including immune evasion, metabolic reprogramming, tissue invasion, and metastasis [1, 2, 3]. Cancer‐associated fibroblasts (CAFs) differ from fibroblasts in healthy tissue, driven by complex reciprocal interaction with cancer cells [3, 4, 5, 6]. Under the influence of the cancer microenvironment, CAFs can adopt a chronically activated alpha‐smooth muscle actin (α‐SMA)‐expressing, contractile myofibroblast phenotype, comparable to the transient reversible phenotype adopted by fibroblasts in the wound‐healing process. CAFs typically produce more ECM and ECM remodelling proteins, and have higher rates of proliferation than normal resident, and apparently quiescent fibroblasts [5]. In contrast to myofibroblasts involved in wound healing, CAFs may have limited ability to reacquire a quiescent state and can display resistance to apoptosis. CAFs are thus distinct from myofibroblasts acquired in acute and chronic inflammation [7].

Over recent years, a more complex picture has emerged as mesenchymal cell markers have been better defined, fuelled by the development of single‐cell transcriptomic and proteomic technologies. This has revealed dramatic CAF heterogeneity, with distinct subpopulations playing diverse and often conflicting roles in the regulation of tumour biology. In addition to the classically recognised α‐SMA^high^ myofibroblast phenotype CAFs, a range of CAF subsets associated with immune modulation have been identified. Distinct CAF populations show variable expression of classical fibroblast markers such as α‐SMA, fibroblast activation protein (FAP), and podoplanin (PDPN). Some CAF subtypes or phenotypes appear to be interconvertible, while others appear restricted to distinct lineages. This necessitates a reappraisal of the source of distinct CAF subtypes and their evolving roles as tumours develop and respond to treatment.

## Conflicting results from early attempts to target CAFs therapeutically

Numerous studies report that CAFs promote cancer cell growth, survival, invasion, and drug resistance. Elevated α‐SMA expression is causatively associated with enhanced contractility, which can promote migration and tissue invasion [8, 9]. Paracrine production of growth factors and cytokines, including hepatocyte growth factor (HGF), insulin‐like growth factor (IGF), and stromal‐derived factor (SDF‐1/CXCL12) support a protumourigenic, chemotherapy‐resistant and immune‐suppressive environment in a variety of cancers [6, 10, 11, 12, 13, 14, 15, 16]. Pancreatic cancer models provide the most exhaustive preclinical rationale for the central role for CAFs in tumour biology. Supporting a role in metastasis, CAFs have been shown to directly lead cancer cell invasion by generating tracks through the ECM for cancer cells to collectively migrate and invade [17]. Further, the identification of pancreatic stellate cells (PSCs) at secondary metastatic sites in implantation murine pancreatic ductal adenocarcinoma (PDAC) models with gender mismatch implicates myofibroblasts directly in the distant spread of disease [18]. In PDAC, the initial indication that targeting stromal myofibroblasts might improve therapy response came from transgenic mouse models where inhibition of hedgehog signalling with IPI‐926 depleted the α‐SMA+ stroma to enhance vascularisation and gemcitabine penetration (perfusion) and, thus, response [19]. A variety of agents limiting CAF or PSC function in diverse preclinical models have now been shown to improve gemcitabine or immunotherapy responses for PDAC, including FAK inhibitors (VS‐4718), vitamin A analogues (all trans‐retinoic acid), and vitamin D receptor agonists (calcipotriol) [20, 21, 22, 23, 24].

In stark contrast to these promising results, efforts to suppress pathological CAF functions, by depleting α‐SMA‐positive fibroblasts or prevent stromal activation by targeting hedgehog signalling in multiple transgenic murine models of PDAC resulted in more aggressive, faster‐progressing metastatic disease [25, 26, 27]. Furthermore, rather disappointingly, a Phase Ib/II clinical trial using hedgehog inhibitors (IPI‐926) to block fibroblasts activation in combination with gemcitabine alone in pancreatic cancer patients was also terminated early due to disease acceleration (NCT01130142) [28]. Studies have additionally shown that impeding myofibroblast differentiation or functional phenotype in transgenic and implantation mouse models can result in more invasive inflammatory tumours [29, 30]. Taken together, these data imply that the initial induction of myofibroblast CAFs (myCAFs) in response to malignant lesions may represent a tumour‐suppressive response to limit cancer development through a variety of mechanisms. However, as tumours evolve this restraining role may be subverted to support invasion and metastasis. Hence, the timing and approach to modulate CAF behaviour as well as understanding how distinct subpopulations of CAFs contribute to favourable or unfavourable behaviour is of critical importance. Furthermore, understanding how these CAF subpopulations evolve with tumour progression and in response to treatment will be critical if we are to intervene clinically with success.

The ability of resident tissue fibroblasts to suppress malignant growth has long been established, and efforts to reprogram activated CAFs to their preactive quiescent state has shown sufficient promise to support clinical trials [21, 23, 31, 32](NCT03520790). Treatment with vitamin A analogues or vitamin D receptor agonists to promote PSC quiescence, based on their physiological responsiveness to these nutritional stores, can suppress oncogenic signalling, tumour growth, and enhance chemotherapy response [21, 24, 33, 34]. Linked to this, distinct subpopulations of patient‐derived CAFs have been associated with differential prognosis, defined by their gene‐expression levels [35]. In a transgenic K‐*Ras* ^+/LSLG12Vgeo^;*Trp53* ^lox/lox^;*Elas*‐tTA/*tetO*‐Cre (KPeC) mouse model, a subset of Saa3 (Serum amyloid A apolipoprotein family) null CAFs can suppress cancer growth [36]. Using a combination of human samples and orthotopic KPC (*Pdx1‐Cre;Kras* ^LSL−G12D/+^ *;p53* ^fl/+^)‐derived murine models, as well as lineage tracing, a subpopulation of tumour restraining CAFs expressing Meflin (mesenchymal stromal cell‐ and fibroblast‐expressing Linx paralogue; a glycosylphosphatidylinositol‐anchored protein) have also been described in PDAC [37], which appear to cause alterations to collagen matrix layout. Lineage tracing also suggested that reprogramming of Meflin‐positive PSCs or CAFs to α‐SMA^high^ CAFs that are both positive and negative for Meflin expression may contribute to CAF functional heterogeneity in tumours. Screening for chemicals capable of promoting conversion of tumour promoting CAFs to rCAF identified the synthetic retinoid Am80 (Tamibarotene) as a promising candidate. Am80 upregulates Meflin expression in stromal cells and enhances gemcitabine chemotherapy response in a subcutaneous mT5 (KPC‐derived PDAC cell line) mouse PDAC model [38], and is now being trialled clinically in combination with gemcitabine and nab‐paclitaxel (Phase I/II; NCT05064618) [39]. Together these diverse studies suggest subpopulations of CAFs can exhibit both tumour‐suppressor and ‐promoter functions at distinct stages of disease development.

A number of mechanisms may contribute to CAF tumour‐suppressive functions, including suppression of inflammation and deposition of tumour‐restraining matrix [2, 40, 41, 42]. For example, α‐SMA+ myofibroblast‐specific deletion of *Col1a1* using a dual recombinase FSF‐Kras^G12D/+^;Trp53^frt/frt^;Pdx1‐Flp (KPPF); α‐SMA‐Cre;R26^Dual^ transgenic mouse model resulted in CXCL5 upregulation in cancer cells, leading to augmented recruitment of CD206 + Arg1+ myeloid‐derived suppressor cells (MDSCs), which in turn suppressed CD8+ T cells, leading to aggressive tumours [43]. This could be at least partially reversed by combined targeting of CXCR2 and CCR2, demonstrating a critical role for collagen and CAFs in the orchestration of the tumour microenvironment (TME) [43]. It remains to be seen whether the inherent differences between tumour‐promoting and tumour‐suppressing CAFs is spatially restricted within the juxta‐tumoral space as opposed to the pan‐stromal space [44], as well as temporally regulated as the tumour evolves: an aspect which will be explored by emerging spatially resolved sc‐RNAseq and lineage tracing.

## CAF heterogeneity and plasticity

Dichotomous roles for fibroblasts in tumour development have long supported the premise that distinct subpopulations of CAFs may modulate tumours differentially [4]. The coexistence of diverse fibroblasts has also been long appreciated [45]. In 2011, Kiskowski *et al* elegantly demonstrated, for the first time, that mixed populations of transforming growth factor‐beta (TGF‐β) responsive and TGF‐β nonresponsive stromal cells can drive prostate adenocarcinoma, whereas alone these stromal components only support benign or precancerous lesions [46].

### Myofibroblast CAF and inflammatory CAF switching

**Figure 1:**
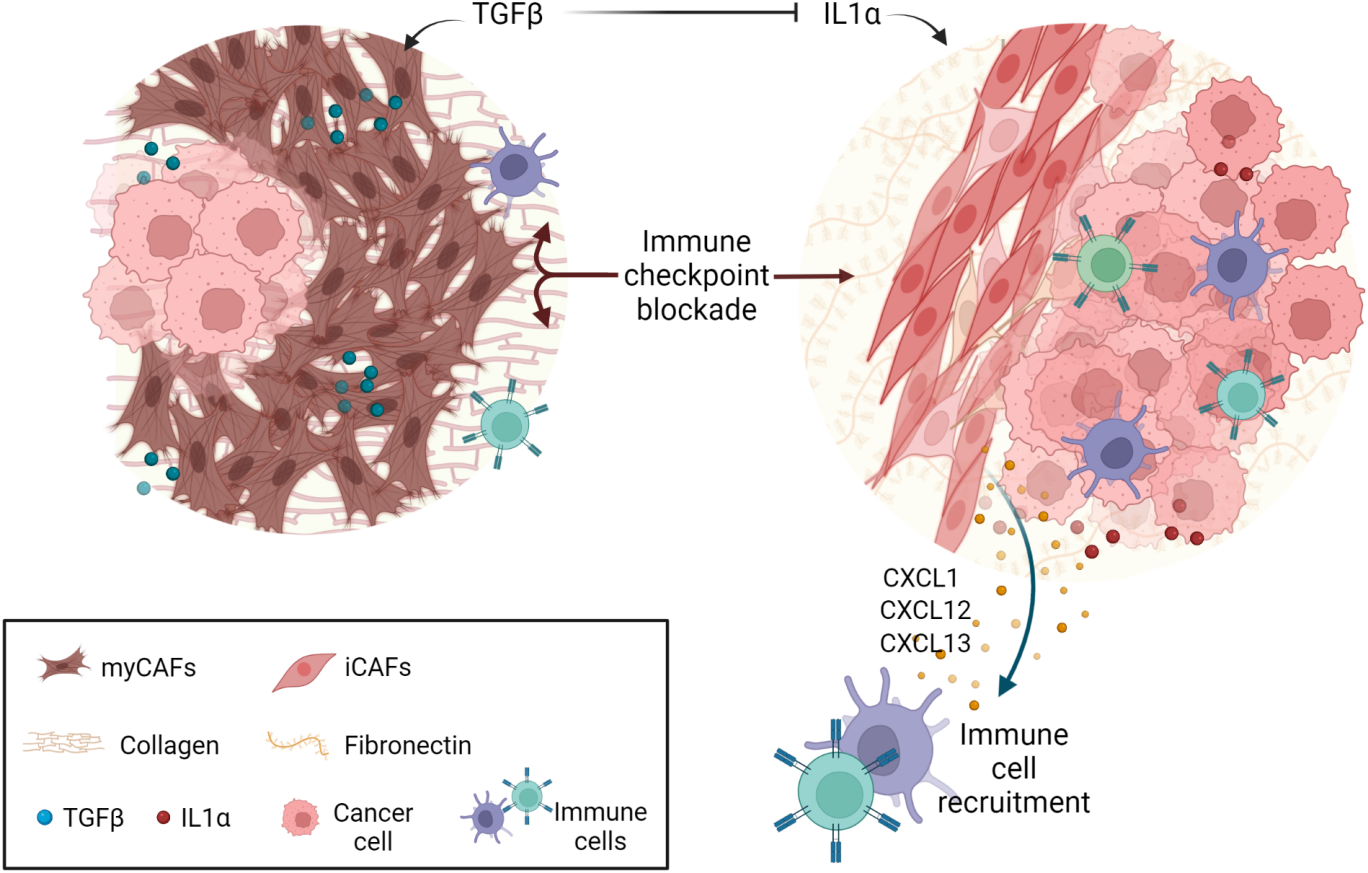
iCAFs and myCAFs in cancer progression. Myofibroblast‐CAFs and inflammatory‐CAFs are common to diverse solid tumours. Their activities are polarised and antagonised by TGF‐β and IL1 signalling to promote distinct aspects of tumour biology, including ECM signatures, immune infiltrate, and malignant cell phenotypes. Created with BioRender.com.

Öhlund *et al* took the CAF dichotomisation a step further by defining two key interconvertible spatially resolved subpopulations of myofibroblast CAF (myCAF) and inflammatory CAF (iCAF) in both KPC mouse and human pancreatic cancer (Figure 1) [47]. MyCAFs broadly represent a classical TGF‐β activated subtype, expressing high levels of the classical CAF markers α‐SMA and FAP, and are found proximal to malignant cells. In contrast, iCAFs are found distal to tumour cells within the stroma and display low α‐SMA expression with upregulation of JAK/STAT and nuclear factor kappa B (NFκB ) signalling, accompanied by secretion of inflammatory cytokines including interleukin‐6 (IL‐6), leukocte inhibitory factor (LIF), and CXCL1 [29, 47]. MyCAF and iCAF phenotypes were readily interconvertible *in vitro*, dependent on culture conditions, and signalling pathway modulation [30, 47, 48]. The interplay between contractile α‐SMA^high^ CAFs and secretory/immunomodulatory CAFs has emerged as a unifying theme across many solid cancer types (Table 1).

**Table 1:** Cancer‐associated fibroblast subtypes and markers.

CAF subtypes	Selected *gene/*protein markers	Biological functions/notes	Cancer type/model	Study type(s)	References
**Pancreatic cancer**					
myCAFs	*Acta2, Ctgf, Postn*	TGF‐activated	Human PDAC/KPC mice	RNAseq sc‐RNAseq	[29, 47, 49]
iCAFs	*Il6, Cxcl1, Cxcl12, Ccl2, Pdgfra, Has1*	Il1‐activated. Promote inflammation			
apCAFs	*H2‐Ab1*, (MHC class II) *Cd74* (CD74), *Saa3*	Antigen‐presentation/T‐cell activation	KPC mice		[50, 66]
Subtype A	*POSTN*/POSTN	Poor outcome	Primary pancreatic CAFs	NanoString nCounter	[35]
Subtype B	*MYH11*/MYH11, *ACTA2/*ACTA2	Intermediate outcome			
Subtype C	*PDPN*/PDPN*, ACTA2/*ACTA2	Immune. Good outcome			
Subtype D	*ACTA2/ACTA2*	Prognostic and phenotypic			
Lrrc15(+)	*Lrrc15(+), Cd105 (ENG), Serpine2, Col15a1*	Tumour promoting myCAFs. TGF‐β promoted phenotype.	Human PDAC/KPP mice	sc‐RNAseq	[65]
Dpp4+	*Dpp4, Ly6c1, Pdgfra*	Inflammatory CAFs. IL1 promoted			
Mesothelial	*Cd74, H2‐Ab1, Pdpn, Dpp4*	Mesothelial/apCAF related			
Cd105^pos^	*Cd105 (ENG), Postn, Cxcl14, Col6a1*	Tumour permissive CAFs. TGF‐β response	KPC mice and multiple GEMM cancer models	Multi‐omics	[64]
Cd105^neg^	*Cd74, H2‐Ab1, Cxcl2, Gas1, Saa3*	CD105^neg^ – antitumour immunity/tumour suppressive			
	Shared: *Pdpn, Vim, Col1a1, Col1a2, Acta2*				
rCAFs	MEFLIN/Meflin	Tumour restraining (rCAFs). Suppress poor‐differentiation	Human PDAC and KPC/Meflin‐KO mice	IHC/ISH	[37, 51]
pCAFs	ACTA2high	Tumour promoting (pCAFs)			
**Breast cancer**					
vCAFs	*Vegfa*, *Nid2*	Vascular CAFs ‐ Vascular development/ angiogenesis. Perivascular origin.	MMTV‐PyMT mouse model	sc‐RNAseq	[73]
mCAFs	*Fbln1*, *Pdgfra Dcn, Vcan, Col14a1, Cxcl14*	Matrix production/fibrosis. From resident fibroblasts. Decrease during progression			
dCAF	*Scrg1*	Malignant cell EMT			
myCAFs	*Acta2, Lrrc15*	TGF‐β activated myCAFs	Subcutaneous 4T1 Breast cancer model	sc‐RNAseq	[52]
iCAFs	*Il6, C3, Cd34, Dpp4* (CD26), *Ly6c1* (Ly6C)	Inflammation and immune cell regulation/recruitment			
vCAFs	*Vegfa, Acta2, Lrrc15*	Vascular CAFs ‐ Vascular development/ angiogenesis			
ilCAFs	*CD74+*	Interferon licenced CAFs. Induced on TGF‐β blockade			
myCAFs,	*ACTA2, PDPN, FAP*	TGF‐β activated myCAFs	Primary breast tumours		[53]
iCAFs	*CD34, CXCL1,CXCL12, CXCL13*	Inflammation and immune cell regulation/recruitment			
CAF‐S1	FAP, FSP‐1, ACTA2, CD29	Subsets of CAF‐S1 include myCAFs (ecm‐myCAFs and TGF‐β‐myCAFs) and iCAFs. myCAFs immunosuppressive	Human primary breast cancer	FACs sorted sc‐RNAseq	[48, 54]
CAF‐S2 and S3	CD29	Normal tissue fibroblast signature			
CAF‐S4	CD29, ACTA2	Cancer‐associated			
**Selected other cancers**					
myCAFs	*ACTA2, HAS2*	myCAFs promote Has2/HA axis Hepatic Stellate Cell Origin	Cholangiocarcinoma (murine and human ICC)	sc‐RNAseq	[55]
iCAFs,	*HGF, cytokines, chemokines*	iCAFs promote growth through HGF. Hepatic Stellate Cell Origin			
mesoCAF		Mesothelial CAFs			
myCAFs,	*ACTA2, COL1A1*	Myofibroblasts	Gastric cancer	sc‐RNAseq	[56]
iCAFs	*CXCL12, IL6* and *CXCL14*	iCAFs regulate T‐cells			
eCAFs (ECM)	*MMP14*, *LOXL2*, and *POSTN*	proinvasive ECM regulating/M2 macrophage interaction			

The emergence of single‐cell technologies has now revealed huge diversity and plasticity within these broad iCAF and myCAF categories, and have also uncovered the signalling networks governing their distinct cellular states. Among the key cytokines, TGF‐β and IL1α influence fibroblast phenotypes in opposing ways to polarise and generate the myCAF and iCAF populations in multiple tumour types [29, 49, 52, 64]. The inflammatory FAP^high^ phenotype, while dominant at the early phases of tumour development, appears to gradually give way to a more myofibroblastic α‐SMA+ CD34^neg^ phenotype that is contractile and produces a stiff collagen‐rich matrix as the disease progresses in transgenic KPC and KPP murine models [42, 65]. Mechanistically, TGF‐β downregulates IL1R1 expression and thus suppresses the more secretory iCAF phenotype [29], which likely contributes to the spatial resolution of these cell types in tumours, where iCAFs are comparatively distal to the TGF‐β‐producing malignant epithelium [47]. Typically, FAP^high^ α‐SMA^low^ iCAFs are associated with increased tumour progression [29, 49, 57].

Strikingly, uncoupling myofibroblast functionality can also result in a switch from a myCAF expression signature to an iCAF signature in KPC‐derived syngeneic orthotopic pancreatic tumours [30]. Loss of the Rho‐effector kinase protein kinase N2 (PKN2) from PSCs *in vitro* suppressed cell contractility and mechano‐sensing, while promoting adoption of an iCAF‐like matrisome and expression of the iCAF markers IL6 and LIF. *In vivo*, stromal deletion of PKN2 also resulted in a shift from myCAF to iCAF signatures in orthotopic murine tumours, accompanied by enhanced EMT and IL6‐JAK‐STAT3 signalling. This implies that the role for PKN2 in myofibroblast function delineated in PSCs is conserved in CAF populations in orthotopic tumours. Similarly, targeting FAK in a subset of FSP‐positive CAFs―which would also suppress mechanotransduction‐mediated myofibroblast activation― likewise resulted in more aggressive PDAC tumours, accompanied by enhanced inflammatory chemokine signalling and a switch in tumour metabolism towards malignant cell glycolysis [58]. These results tightly concur with the concept that CAFs exist in interconvertible states, but also highlight that targeting one pathological function can result in bias towards distinct CAF subpopulations, with unexpected and sometimes undesirable consequences.

### Many functionally distinct subpopulations of CAFs continue to emerge

Sc‐RNA data reveals abundant fibroblasts, endothelial cells, pericytes, mesothelial cells, and immune cells as major stromal cell types in pancreatic tumours [59, 65]. Historically, a lack of clearly defined markers has hampered isolation and definition of CAFs and their various subtypes. Classical markers such as α‐SMA, vimentin, FAP, PDGFRα, and PDPN [9, 50, 60, 61, 62] have been useful, but their high‐level expression by other cells types, such as pericytes, and heterogeneous expression across fibroblast subpopulations can confound deconvolution. Single cell‐RNA sequencing (sc‐RNAseq) of total tumour cell populations, coupled with focussed sc‐RNAseq of fibroblast‐enriched fractions, has resolved this problem by defining highly discriminatory stromal cell signatures. Importantly, sc‐RNAseq data can also be used to infer CAF subpopulation functions and heterocellular interactions. Lineage tracking and temporal analysis has revealed diverse cell origins, differentiation trajectories, and variable interconvertibility between CAF subtypes. Despite significant heterogeneity within tumours, functionally distinct CAF subtypes identified are significantly conserved across distinct cancer types (Table 1).

In pancreatic cancer, multiple sc‐RNAseq studies have now mapped CAF populations in mouse models and human primary tumours. Sc‐RNAseq studies by Tuveson's group characterised iCAFs and myCAFs, and identified a novel class of MHC class II and CD74 expressing ‘*antigen presenting*’ CAFs in KPC mouse and human tumours; KPC‐derived apCAFs were able to activate CD4+ T‐cells in an antigen‐specific manner, in keeping with a role in tumour immune surveillance, at least in the KPC murine model [66]. Although apCAFs appear related to myCAFs in the KPC model, a mesothelial origin for apCAFs has been also been proposed in single‐cell studies [64, 65] (See Box 1). Leucine‐Rich Repeat Containing 15 (LRRC15) expression was identified by Dominguez *et al* as a defining feature of CAFs over normal tissue fibroblasts in both *Pdx1* ^cre/+^;*LSL‐Kras* ^G12D/+^;*p16/p19* ^flox/flox^ (KPP) mice and human pancreatic cancer patients [65]. CD105, an auxiliary receptor within the TGF‐β signalling pathway [80] additionally defines a precursor fibroblast population found in normal tissue [65]. Elegant pseudo‐time analyses suggest that CD105+ resident fibroblasts give rise to LRRC15^high^ myCAFs as tumours become established and progress, whereas an alternative lineage of CD105^neg^;DPP4^+^ resident fibroblasts give rise to iCAFs. CAFs derived from both lineages show high‐level expression of *Col1a1* and *Col1a2* [65]. In contrast, analysis of human samples suggests that iCAFs and myCAFs can derive from a single CD105+ lineage, polarised to IL1 or TGF‐β activated states, urging caution when extrapolating from mouse models [65]. Differences may also reflect the pan‐pancreas tumour development in these transgenic mouse models, as opposed to solitary tumour focus in humans.

**Box 1:** CAF origins and lineages.

CAFs have been reported to originate from many sources, including resident fibroblast populations, mesenchymal stem cells (MSCs) and transdifferentiation of distinct stromal populations such as adipocytes pericytes and mesothelial cells (reviewed in [1, 3, 63]). Additionally, distinct CAF subtypes appear to be dynamic and interconvertible, epitomised by spatial and cytokine regulation of iCAF and myCAF state in many disease settings. In pancreatic cancer, the existence of noninterconvertible fibroblast subtypes such as CD105^pos^ and CD105^neg^ populations, both in tumours and the healthy pancreas, indicates that distinct lineages are likely to contribute to heterogeneity [64, 65]. Interestingly, CD105^pos^ and CD105^neg^ fibroblast populations can be found in distinct localisations in the normal pancreas and gene expression suggests a developmental link between CD105^neg^ fibroblasts and mesothelial cells [64]. Dominguez *et al* provide additional evidence that iCAFs may largely derive from a CD105^neg^ resident population [65]. That study also provided evidence that CD74 and H2‐Ab1‐expressing CD105^neg^ fibroblasts‐equivalent to the apCAFs defined by Elyada *et al* [66]‐have a mesothelial origin, although the origins of apCAFs may be distinct in KPC and human PDAC [65, 66]. Mesothelial to mesenchymal transition has been reported in other pathological tissue fibrosis [67]. Garcia *et al* reported distinct Gli1 and HoxB6 fibroblast lineages in the healthy mouse pancreas, which can both contribute significantly to KF (Ptf1aFlpO/+;KrasFSF‐G12D/+) and KPF (Ptf1aFlpO/+;KrasFSF‐G12D/+;Trp53FRT‐STOP‐FRT/+)‐driven tumour CAFs, with Gli1+ cells dominating [68]. These distinct resident‐fibroblast lineages, alongside lineage tracking, challenge the concept that pancreatic CAFs are predominantly derived from PSCs [69, 70, 71]. In fact, recent lineage tracing studies from the Sherman group indicate that PSCs appear to contribute only a minor subpopulation of CAFs in both KPC mouse orthotopic and human PDAC tumours [71]. Non‐PSC pancreatic fibroblasts can expand into abundant α‐SMA^high^ CAFs in PDAC. This study also identified unique nonredundant functions associated with CAFs from distinct origins [71]. In the PyMT‐MMTV breast cancer model [72], distinct CAF populations also appeared to derive from distinct resident mesenchymal lineages. Both resident tissue fibroblasts and mesenchymal cells with a perivascular origin contribute to tumour CAFs, which vie for dominance as tumours progress [73]. These studies highlight diverse lineage origins even within resident mesenchymal populations, with the caveat that differences between primary human tumours and mouse models are likely to exist. The importance of MSCs as a source of CAFs appears to vary considerably between tumour types. In adoptive transfer experiments, bone marrow‐derived cells were shown to contribute up to 25% of fibroblasts in a large‐T‐driven model of pancreatic insulinoma, as well as contributing significantly to myofibroblasts in many tissues [74, 75, 76]. MSC‐derived CAFs in breast cancer are also functionally distinct from resident fibroblast‐derived CAFs, showing no expression of PDGFRα and associating with worse prognosis [77]. Interestingly, in a study of secondary tumours arising in sex‐mismatched bone‐marrow transplant recipients, the majority of α‐SMA+ CAFs were recipient‐derived [78]. In colorectal cancer most CAFs appear to be derived from resident pericryptal fibroblasts [79]. Similarly in pancreatic cancer, resident mesenchymal populations appear to contribute the majority of CAFs, despite distinct lineage sources [64, 65, 68, 70]. Finally, EMT of malignant cancer cells also contributes to α‐SMA^high^ CAF‐like populations in tumours [73]. Though these cells are not classically considered CAFs, they are certain to contribute significantly to ECM and immune regulation, in addition to the well‐documented role of EMT in migration, invasion, and metastasis. As distinct origins translate into distinct tumour‐regulating functions, it is perhaps unsurprising that targeting ubiquitous CAF regulators can lead to unpredictable outcomes.

Following this theme, Hutton *et al* used a combination of mass cytometry and transcript analysis to identify CD105 as a key CAF lineage marker defining tumour suppressive CAFs (CD105^neg^), which act by supporting antitumour immunity [64]. Intriguingly, KPC‐derived CD105^pos^ and CD105^neg^ CAFs were not interconvertible, but both could adopt either an myCAF or iCAF phenotype, potentially indicating multiple origins for these phenotypic classifications [64, 65]. In line with Dominguez *et al*, it is proposed that CD105^pos^ and CD105^neg^ CAFs derive from distinct spatially resolved precursor fibroblasts and provide evidence that CD105^neg^ precursors may be related to mesothelial lineages. The contribution of distinct fibroblast lineages in human PDAC is a hot topic for further exploration (See Box 1).

Some consensus is beginning to emerge for the categorisation of functionally conserved CAF categories. Meta‐analysis of human sc‐RNAseq data from multiple cancer types defined six pan‐CAF subtypes and associated markers equating to myCAFs, iCAFs (pan‐iCAF and pan‐iCAF‐2), dCAFs (ECM producing), nCAFs (normal fibroblast‐like signature), and pCAFs (proliferating) [81]. Signatures derived from these pan‐CAFs had different prognostic power between distinct cancer types [81]. In addition to the widely described myCAF and iCAF subcategories, CAFs dedicated to ECM regulation and proliferating CAF signatures have also been widely identified in mouse and human datasets (Table 1) [35, 52, 56, 73, 82].

While detailed maps of the CAF landscape have been provided for mouse models and a small number of primary human PDAC tumours, less has been done to examine interpatient and intrapatient variability in CAF populations. Transcriptomic analysis of primary CAF isolates from PDAC patients defined at least four distinct CAF subgroups with differing expression of known fibroblast identifiers and with a distinct impact on patient prognosis. For example, enrichment with α‐SMA^high^ ECM+ myofibroblast CAFs (pCAFassigner subtypes B and D) was associated with a worse prognosis, while α‐SMA^low^ immunomodulatory CAF (pCAFassigner subtype C) signatures predicted better outcome when interrogated across ICGC and TCGA datasets [35]. Lee *et al* also conducted sc‐RNAseq on primary tumour extracts, including metastases to identify tumour subtypes and heterogeneous TME responses and CAF content, which identified potential immunotherapy vulnerabilities [83]; high apCAF abundance relative to other CAF subtypes, was associated with low Teffector/Tregulatory (T_eff_/T_reg_) ratios, supporting a key role for CAF ratios in regulation of antitumour immunity. This study also highlighted the existence of multiple epithelial cancer subtypes within individual tumours, and metastases, independent of the classification system used [83]. Taking this a step further, Grünwald *et al* use spatially resolved multi‐omics to powerfully demonstrate that CAF differentiation trajectories in subTME regions of PDAC tumours dictate localised tumour immunity, cancer cell phenotype, and treatment susceptibility [84]. Similar phenotypically distinct ‘tumour glands’ exhibiting distinct CAF‐cancer cell relationships have also been reported by Ligorio *et al* [85]. Both studies highlight localised and heterogeneous intratumoral evolution of cancer‐CAF relationships in primary human PDAC.

In summary, resident fibroblasts from distinct lineages most likely contribute to functionally distinct CAF populations as tumours evolve, with early subtypes (such as pCAFassigner subtype A [35]) being more pliable and later subtypes being more resistant to interconvertibility. Some CAFs may arise as a cause rather than a consequence of PDAC evolution [84, 85] (See Box 1). In the next sections we will address the integral role that fibroblasts play in immune cell recruitment and whether CAFs can be manipulated to support specific therapeutic interventions.

## Manipulating CAF populations to promote therapy response

**Figure 2:**
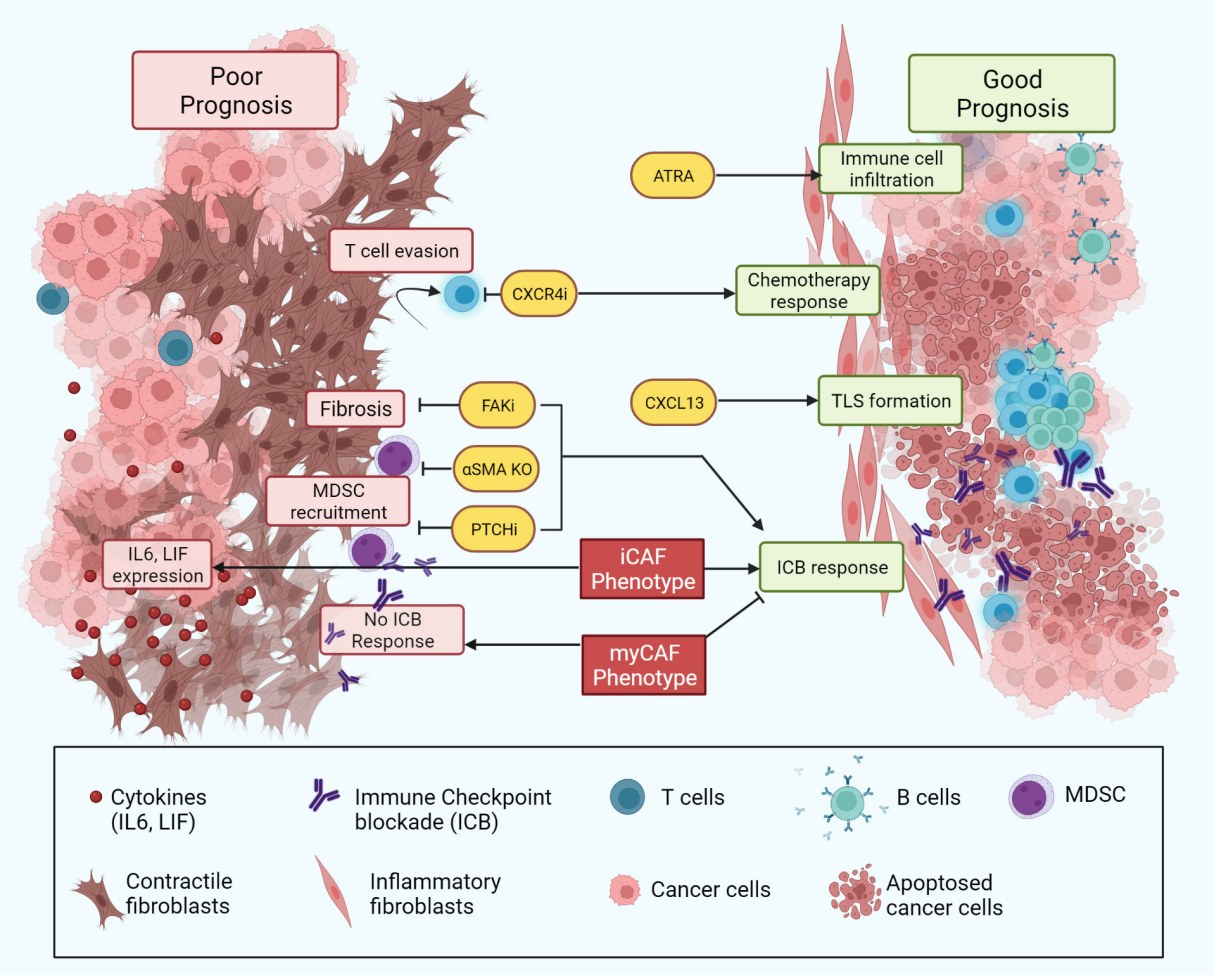
CAF modulation of the immune microenvironment and immunotherapy. Different CAF subtypes have distinct tumour‐promoting and tumour‐suppressor functions. myCAFs can have both tumour‐restraining but also support an immune‐suppressive microenvironment that can block immunotherapy response. iCAFs produce inflammatory mediators and chemokines that can drive aggressive tumours with high EMT gene‐expression signatures. While iCAFs can also support immunosuppression, enhanced inflammation and immune cell recruitment can also support enhanced immunotherapy response. Created with BioRender.com.

Immune evasion is a key hallmark of cancer [86]. While initial efforts to target CAFs focussed on improving chemotherapy responses, opening up tumours to the immune system has now become a key focus (Figure 2). CAFs play a key role in the recruitment and maintenance of immune cells in solid tumours, and are considered the architects of the immune suppressive environment. Importantly, immune engagement can improve the prospects for immunotherapy and chemotherapy responses, and support enduring antitumour protection.

CAFs can bias T‐cell responses through a variety of mechanisms, including: (1) exclusion of cytotoxic CD8+ T‐cells [42, 44, 87, 88] by spatially restricting them to pan‐stromal space as observed in human samples, and (2) promotion of T‐regulatory cells as seen in murine models with interference of hedgehog signalling [29, 49]. Evolution of cancer from precursor lesions to invasive disease is associated with progressive loss of effector T‐cells and enhanced MDSC content, choreographed by progressive immunomodulatory CAF evolution [89]. However, Pancreatic intraepithelial neoplasia (PanIN) resolution in humans cannot and has not been studied to lend credence to the hypothesis that stromal‐ and immune‐modulation is an active program. The key determinants of immune evasion are the presence of immune‐suppressive cytokines and recruitment of immune‐suppressive myeloid cells, biasing the immune response towards a more regulatory phenotype. Sc‐CAF mouse studies have consistently shown CAF production of immunomodulatory cytokines such as Il6, Cyr61, and Cox‐2, which can create immune suppressive environments that diminish the activity of effector immune cells. CAF‐derived chemokines like Cxcl1, Cxcl12, and Cxcl2 also recruit a heterogeneous population of largely immunosuppressive or tumour‐promoting myeloid‐derived cells, including monocytes, macrophages, neutrophils, and MDSCs, at least in murine studies [41, 42, 65, 89, 90].

The unfavourable immunosuppressive impact of myeloid and lymphocyte immune cell infiltration on tumour growth may nonetheless be manipulated towards a favourable response to immunotherapy (Figure 2). Inducing a stronger inflammatory CAF phenotype in tumours has improved the response to immunotherapy and/or chemotherapy in murine models [25, 42, 52, 65]. This provides evidence that manipulating distinct subpopulations of CAFs might hold the key to engaging immunotherapy in pancreatic and other solid cancers. Enhanced tumour inflammation has however been associated with more aggressive tumour growth in mouse PDAC models and worse prognosis in human datasets, and should be cautiously approached [29, 30, 40, 91, 92, 93, 94]. A balance of interventions must be found to concomitantly impede tumour progression, promote antitumour immunity, and enhance therapy efficacy.

### Targeting the immune suppressor CAF function

Evidence that CAFs regulate antitumour immunity was provided by Kraman *et al*, who showed that ablation of FAP+ stromal cells promoted tumour‐antigen‐specific immune clearance of lung and pancreatic cancer murine models [2]; this has since been confirmed in a murine KPC PDAC model [95]. More recently, Özdemir *et al* demonstrated that targeted depletion of immunosuppressive α‐SMA+ CAFs in a genetic PDAC model (PKT: *Ptf1a* ^cre/+^;*LSL‐Kras* ^G12D/+^;*Tgfbr2* ^flox/flox^) resulted in reduced fibrosis and accelerated tumour growth [25]; this was, however, accompanied by significant sensitisation to anti‐CTLA‐4 immunotherapy, with survival dramatically prolonged in comparison to non‐CAF‐depleted controls. This sensitisation was associated with enhanced T_eff_/T_reg_ ratios and CTLA‐4 expression and exemplifies the coexistence of protumoural roles with promotion of therapy response.

The CXCL12/CXCR4 axis also shows significant promise as a mechanism for reducing CAF‐mediated fibrosis and enhancing checkpoint inhibitor response in both KPC pancreatic and orthotopic breast cancer murine models (Figure 2) [42, 96]. This is also demonstrable in human samples with activated PSCs orchestrating this signalling [44], and this axis is now being targeted in clinical trials (NCT04177810, NCT02907099). In KPC mice, the inhibition of focal adhesion kinase (FAK) also reduces tumour fibrosis and immunosuppressive cell infiltration (MDSCs, tumour‐associated macrophages and T_regs_) resulting in enhanced response to checkpoint blockade (anti‐PD1 and anti‐CTLA4) and chemotherapy [22]; in this model, tumour‐cell intrinsic FAK appears to be the key driver of CAF expansion, tumour fibrosis, and immune suppression, in contrast to the previously described CAF intrinsic mechanisms. Stromal normalisation with the vitamin A analogue ATRA can enhance CD8+ T‐cell recruitment to PDAC tumours and clinical trials are underway in combination with chemotherapy [23, 44]. The impact of ATRA on checkpoint blockade remains to be assessed. In contrast, *in vitro* evidence might suggest that vitamin D agonists could suppress T‐cell responses, although *in vivo* validation and combination with checkpoint inhibitors is currently lacking [97].

Hedgehog (Hh), an overexpressed protein in pancreatic cancers, appeared to be a promising target for treatment [19]. Despite early preclinical promise, targeting the hedgehog pathway inhibition with vismodegib has failed to improve chemotherapy responses in early clinical trials [98, 99]. Surprisingly, although Hedgehog pathway inhibition has been trialled with additional targeted therapies against mammalian target of rapamycin (mTOR) or epidermal growth factor receptor (EGFR) pathways [100, 101], combinations of hedgehog targeting with immunotherapy has been limited; a single clinical trial has recently begun combining the hedgehog pathway inhibitor NLM‐001 with zalifrelimab (anti‐CTLA‐4) and chemotherapy (NCT04827953). In support of this approach, Patched 1‐interacting peptide, which inhibits hedgehog signalling, reduced fibrosis, enhanced CD8+ T‐cell infiltration, and augmented anti‐PD1 response in mice [102]. Cotargeting the hedgehog pathway alongside CXCR4 also improved gemcitabine response in an orthotopic pancreatic model, which may be linked to CXCR4 regulation of antitumour immunity [42, 96, 103]. Further exploration of the hedgehog pathway in combination with immunotherapy is certainly warranted.

The immunosuppressive role of TGF‐β has been examined in a variety of solid tumours, including PDAC [52, 65, 104, 105]. TGF‐β appears to drive an immune‐suppressive myCAF landscape, with a poor response to checkpoint blockade. The response to anti‐PDL1 treatment has been found to be diminished in human tumours across multiple cancer types enriched with TGF‐β driven LRRC15+ myCAFs [65]. Likewise, in metastatic urothelial cancers, the lack of response to anti‐PD‐L1 therapy was strongly associated with a TGF‐β gene expression signature, indicating a possible role of fibroblasts in therapy resistance by sequestering CD8+ T cells in collagen and fibronectin‐rich peritumoral stroma in patient samples. Furthermore, anti‐TGF‐β and anti‐PD‐L1 combine to provoke antitumour immunity and tumour regression in a mouse EMT6 mammary carcinoma model [104]. In concurrence, TGF‐β neutralization in a subcutaneous 4T1 implantation model of breast cancer led to diminished myCAFs, enhanced CD8+ T‐cell infiltration, and augmented anti‐PD1 response [52]. Enhanced response was associated with an increase in iCAFs and the emergence of a CD73+ IFN‐γ responsive CAF subtype (interferon‐licenced CAFs, ilCAFs); it currently remains unclear whether the increase in iCAFs and ilCAFs results from reprogramming of existing myCAFs, or through expansion of distinct mesenchymal lineages. Switching of myCAFs to iCAFs *in vitro* provides support for conversion of existing myCAF populations, although lineage tracing will be required to definitively answer this question [29, 30, 47]. In multiple genetic murine models of colorectal cancer, TGF‐β inhibition also induced a potent antitumour immune response and enhanced anti‐PD1‐PDL1 therapy [105].

Kieffer *et al* further defined specific subsets of FAP+ myCAFs in primary breast cancer responsible for immune suppression through association with enhanced FoxP3+ PD1+ T_reg_ cells; importantly, *in vitro* coculture experiments with T‐cells indicate that CAFs must adopt a myCAF phenotype to induce FoxP3, PD1 and CTLA‐4 expression [48, 54]. myCAF subtype but not iCAF subtype signatures are enriched in nonresponder groups in immunotherapy trial data for melanoma and non‐small cell lung cancer (NSCLC), implicating myCAF‐mediated immunosuppression in therapy evasion [48]. These studies make a case for the suppression of myCAF signatures to reduce immune evasion and improve immunotherapy response.

### Are iCAFs desirable or dangerous in therapeutic strategies?

While there is some consensus on the existence of immunosuppressive myCAF populations, the role of iCAF subsets in immune suppression remains less clear. Biffi *et al* identified IL1‐driven JAK‐STAT3 as a key pathway governing iCAF identity, and further demonstrated that IL1 signalling antagonises TGF‐β induction of myCAFs, resulting in the distinct polarised CAF clusters [29]. Importantly, the relative levels of iCAFs and myCAFs in KPC tumours can be modulated by targeting these pathways to assess the impact on tumour biology. Targeting the TGF‐β pathway suppresses myCAFs and enhances iCAF populations, and is associated with more aggressive tumour growth, elevated inflammatory signalling, and EMT [29, 49]. Conversely, targeting JAK or LIF to enhance myCAF populations was associated with less aggressive tumour growth [29, 93]. Perhaps significantly, pharmacological targeting of JAK (JAKi) was associated with both enhanced myCAF/iCAF ratios and enhanced absolute CAF and myCAF numbers [29]; each of these changes may contribute to observed tumour phenotypes. More broadly, LIF and IL6 (key markers and regulators of iCAFs) have been variously associated with aggressive, inflammatory, EMT‐rich poor outcome tumours [40, 91, 92, 93, 94]. This is further corroborated in human tumour data, where inflammatory, EMT, and iCAF signatures are all associated with poor outcome [29, 49]. Similar phenotypic changes can be seen upon induction of an myCAF to iCAF switch by targeting the Rho‐regulated kinase PKN2 to uncouple myofibroblast mechanotransduction [30]. Targeting IL1 signalling to limit iCAFs, has therefore been proposed as a method to suppress aggressive tumour growth [29]. Depletion of iCAFs could provide a therapeutic means to suppress production of tumour‐promoting cytokines and chemokines while promoting the adoption of tumour suppressor myCAFs [29]. In opposition to this, targeting the hedgehog pathway with LDE225 shifts the balance away from myCAFs towards iCAFs and suppresses tumour growth [49]. This approach is at odds with the convincing identification of myCAFs as key immunosuppressive populations that block immunotherapy. Such studies would instead favour the suppression myCAFs in favour of iCAFs to support improved immunotherapy response (Figure 1) [52].

iCAFs, by their definition, remain key mediators of the immune landscape in tumours. Enhanced myeloid cell content, skewing of T_reg_/T_eff_ ratios, and loss of CD8+ cytotoxic T‐cells have all been associated with iCAF‐enriched tumour models, indicating potential immune suppressive roles [29, 49]; here, a combination with appropriate checkpoint blockade may be of value. A PDPN+ immunofibroblast population has been directly associated with formation of tertiary lymphoid structures (TLS) dependent on IL13 and IL22 [106]. PDPN+ pCAFassigner subtype C CAFs have been shown to have an association with good prognosis and an immune‐rich phenotype in human pancreatic cancers [35]. This is important because TLS content and activation status represent prognostic biomarkers for a good outcome and predictive biomarkers for response to immunotherapy in multiple tumour types, including PDAC [107, 108, 109, 110, 111, 112, 113, 114]. In melanoma, PDPN+ CAF networks act as lymphoid organisers through production of TLS‐promoting chemokines and through direct interaction with B cells, to orchestrate antitumour immunity [115, 116]. Further, direct induction of TLSs in an orthotopic KPC pancreatic cancer model by intratumoural injection of CXCL13 and CCL21 has been shown to directly augment chemotherapy response [117]. While myCAF populations harbour key tumour‐suppressor populations, iCAFs are likely to remain important regulators of leucocyte content and antitumour immunity. This complicated picture with conflicting roles for iCAFs and myCAFs reflects heterogeneity within these broad CAF categories, evolving roles during disease progression and inherent differences between tissues and CAFs from distinct origins.

## Perspectives on clinical translation

Success in preclinical models has supported numerous clinical trials combining stromal targeting with established interventions in PDAC and other tumour settings (Table 2). In Phase Ib trials combining ATRA with gemcitabine and nab‐paclitaxel, diffusion‐weighted magnetic resonance imaging (MRI) has provided evidence that ATRA can effectively drive stromal modulation, and stromal expression of FABP5 has been identified as a potential predictive biomarker of disease response [23]. Randomised Phase II trials are underway (NCT03307148). A number of trials targeting the vitamin D receptor on PSCs with paricalcitol or high‐dose vitamin D are also in progress, although initial results suggest no improvement in response rate or survival outcomes [119, 120]. Some limited success, however, has been reported with targeting of the TGF‐β axis in a variety of combinatorial studies. A combination of the TGF‐β receptor I kinase inhibitor galunisertib with gemcitabine in a Phase1/IIb trial for unresectable pancreatic cancer resulted in improved patient survival [128]. Galunisertib trials with the anti‐PD‐L1 antibody durvalumab are also ongoing [121]. The novel bifunctional anti‐PD‐L1/TGF‐βRII targeting fusion protein, SHR‐1701, has also shown early promise in refractory solid tumours, including pancreatic cancer [122, 124]. Despite a wide array of trials, targeting the hedgehog pathway has been largely unsuccessful. Vismodegib did not improve the outcome with either gemcitabine or gemcitabine and nab‐paclitaxel. A combination of the hedgehog inhibitor IPI‐926 with gemcitabine was also discontinued early due to poor results (NCT01130142) [28]. Targeting hyaluron directly in the pancreatic cancer stroma has also been the focus of significant clinical activity and some significant success, but this lies beyond the scope of this CAF‐focussed review [133, 134, 135, 136, 137].

**Table 2:** Clinical trials targeting stroma and CAF‐related pathways in PDAC.

Drug/trial name	Years	Phase	Target(s)	Outcome and associated publications	Type of cancer	Trial ID and references
***Stellate cells targeting***						
ATRA in combination with gemcitabine and nab‐paclitaxel/(STAR‐PAC) trial	2017–2020	Ib	Stroma, particularly stellate cells	Repurposing ATRA for stromal‐targeting in combination with gemcitabine‐nab‐paclitaxel is safe and tolerable. This combination will be evaluated further in a phase II RCT for locally advanced PDAC.	PDAC	NCT03307148 [23]
Paricalcitol + gemcitabine + nab‐paclitaxel	2018–2020	I, II	Vitamin D receptors on pancreatic stellate cells	Primary endpoint is overall survival with 100 patients needed to identify a HR of 0.6. Outcome not published Ongoing	Metastatic pancreatic cancer	NCT03520790 [118]
Paricalcitol in combination with paclitaxel, cisplatin, gemcitabine	2018–2023	II	Vitamin D receptors on pancreatic stellate cells	Primary endpoint: complete response rate the end of cycle 3. Outcome not published Ongoing	Metastatic PDAC	NCT03415854
Pembrolizumab with or without paricalcitol	2017–2020	II	Vitamin D receptors on pancreatic stellate cells	The primary endpoint is the percentage of people progressing at 6 moths while on maintenance therapy. Outcome not published	Stage IV pancreatic cancer	NCT03331562 [119]
Paricalcitol in combination with gemcitabine and nab‐paclitaxel	2020–2024	II	Vitamin D receptors on pancreatic stellate cells	Primary endpoint is PFS at 24 weeks from registration and OS and 18 months post last patient registration. Outcome not published Ongoing	Advanced pancreatic cancer	NCT04617067
Neoadjuvant paricalcitol (single agent)	2017–2021	I	Vitamin D receptors on pancreatic stellate cells	Active, not recruiting Outcome not published	Resectable pancreatic cancer	NCT03300921
Paricalcitol and nivolumab plus gemcitabine and nab‐paclitaxel	2020–2024	Early phase I	Vitamin D receptors on pancreatic stellate cells. PD‐L1, PD‐L2	Active, not recruiting Outcome not published	Resectable pancreatic cancer	NCT03519308
Paricalcitol in combination with 5‐FU /leucovorin plus liposomal irinotecan	2019–2022	I	Vitamin D receptors on pancreatic stellate cells	Active, not recruiting Results not published	Advanced pancreatic cancer that progressed on Gemcitabine	NCT03883919 [120]
Paricalcitol and hydroxychloroquine in combination with gemcitabine and nab‐paclitaxel	2020–2023	II	Vitamin D receptors on pancreatic stellate cells	Primary outcome measures: change from baseline tumour size on cross sectional imaging at 8 weeks. (every 8 weeks) Outcome not published Ongoing, recruiting	Advanced or metastatic pancreatic cancer	NCT04524702
Paricalcitol in combination with abraxane/gemcitabine	2014–2020	I	Vitamin D receptors on pancreatic stellate cells	Primary endpoint: number of adverse events. Outcome not published Completed	Resectable pancreatic cancer	NCT02030860
High‐dose Vitamin D (single agent)	2018–2021	III	Vitamin D receptors on pancreatic stellate cells	Terminated due to COVID‐19 pandemic. Primary outcome measure: blood levels of vitamin D. Outcome not published	Pancreatic cancer	NCT03472833
***TGF‐β targeting***						
***TGF‐β immunotherapy combinations***						
Galunisertib + durvalumab	2016–2019	Ib	TGF‐β receptor + Anti‐PD‐L1 antibody	Clinical activity was limited, but both drugs were well tolerated in 32 patients. Studying this combination in patients in an earlier line of treatment was suggested. Completed.	Metastatic pancreatic cancer: refractory, previously treated with ≤2 regimens (2nd or 3rd line)	NCT02734160 [121]
SHR‐1701 In combination with gemcitabine and paclitaxel in first‐line treatment	2020–2022	Ib, II	Bifunctional PD‐L1 and TGF‐β	Ongoing (Active, not recruiting) Primary outcome measures: PP2D, ORR	Advanced /Metastatic pancreatic Cancer	NCT04624217
SHR‐1701	2018–2021	I	Bifunctional PD‐L1 and TGF‐β	SHR‐1701 showed good safety and tolerability profile with promising antitumour activity in refractory solid tumours	Advanced solid tumours including PDAC	NCT03710265 [122]
NIS793 (with and without spartalizumab in combination with gemcitabine/nab paclitaxel) versus gemcitabine /nab‐paclitaxel alone	2020–2022	II	TGF‐β PD‐L1	Ongoing (Recruiting) Primary outcome measures: PFS Secondary outcome measures: safety, tolerability, antitumour activity, and overall survival Outcome not published	Metastatic PDAC	NCT04390763 [123]
SHR‐1701 In combination with famitinib	2020–2021	II	Bifunctional PD‐L1 and TGF‐β	The combination showed promising activity with well‐tolerated toxicities in patients with advanced pancreatic and biliary tract cancers Out of 7 evaluable PC patients, 2 had stable disease Primary endpoint: ORR Secondary endpoint: DCR (43%), PFS, OS, and safety	Previously treated advanced pancreatic and Biliary cancers	ChiCTR2000037927 [124]
NIS793 + spartalizumab (PDR001)	2017–2021	Ib	TGF‐β PD‐1	Completed Data showed target engagement, and TGF‐β inhibition, supporting the mechanism of NIS793. The combination was well tolerated in patients with advanced solid tumours	Multiple including PDAC	NCT02947165 [125]
***TGF‐β alone or with chemotherapy***						
LY2157299 (galunisertib) in combination with gemcitabine	2014–2015	Ib	TGF‐βR1	Completed Galunisertib + gemcitabine combination had an acceptable tolerability and safety with evidence of efficacy	Metastatic or locally advanced pancreatic cancer.	NCT02154646 [126]
Trabedersen (AP12009) (single agent)	2005–2011	I	TGF‐β2	Completed Trabedersen was associated with significant improvement in overall survival (OS) but not Progression free survival (PFS)	Pancreatic cancer Melanoma Colorectal	NCT00844064 [127]
NIS793 in combination with gemcitabine/nab‐paclitaxel versus gemcitabine/nab‐paclitaxel and placebo	2021–2022	III	TGF‐β	Ongoing (Recruiting) Results not published Primary endpoints: DLTs, OS	Metastatic PDAC	NCT04935359
Vactosertib (TEW‐7197) in combination with FOLFOX	2018–2019	Ib, II	TGF‐β/SMAD	Recruitment status: Unknown Primary endpoint: PFS at 6 weeks Outcome not published	Metastatic PDAC (refractory to gemcitabine and nab‐paclitaxel)	NCT03666832
LY2157299 (galunisertib) + gemcitabine	2011–2016	I,II	TGF‐βRI ALK5	Completed Galunisertib + gemcitabine resulted in improvement of overall survival	Metastatic, unresectable pancreatic cancer	NCT01373164 [128]
PF‐06952229 combination therapy with enzalutamide	2018–2022	I, Ib	TGF‐βR1	Active, not recruiting Outcome not published	Advanced solid tumours (multiple including pancreas)	NCT03685591
***Hedgehog inhibitor combinations***						
NLM‐001 in Combination with gemcitabine and nab‐paclitaxel plus zalifrelimab.	2021–2022	I, II	Hedgehog pathway + CTLA‐4	Primary outcome measures: Objective response rate (ORR): Complete response (CR) + Partial response (PR) Ongoing (Recruiting) Outcome not published	Advanced PDAC	NCT04827953
LDE‐225 (erismodegib) (single agent)	2012–2014	I	Hedgehog pathway	Trial withdrawn due to lack of accrual Outcome not published	Surgically resectable pancreatic cancer	NCT01694589
LDE‐225 (erismodegib) (single agent)	2013–2016	NA	Hedgehog pathway	Trial withdrawn (No accrual) Outcome not published	Surgically resectable pancreatic cancer	NCT01911416
GDC‐0449 (vismodegib) + gemcitabine	2010–2017	II	Hedgehog pathway	Completed Primary outcome: Median percentage of CD44 + CD24 + ESA+ cells from FNAC at 3 weeks versus baseline. Secondary outcome: (CR + PR), PFS, percentage of Grade 3 toxicity. GDC‐0449 and gemcitabine combination was not superior to gemcitabine alone	Metastatic pancreatic cancer	NCT01195415 [98]
GDC‐0449 (vismodegib) in combination with gemcitabine and nab‐paclitaxel	2010–2018	II	Hedgehog pathway	Completed Primary outcome: PFS, and Safety. Secondary outcome: Efficacy as assessed by OS, Tumour response, changes in pancreatic Ca stem cells, and Hedgehog deregulation. Vismodegib + chemotherapy did not improve efficacy as compared with chemotherapy alone.	Metastatic pancreatic cancer	NCT01088815 [99]
IPI‐926 (saridegib) + gemcitabine	2010–2017	Ib, II	Hedgehog pathway	Completed Primary outcome measures: safety profile, and overall survival. Secondary outcome measures: Cmax, PFS, TTP, ORR. The combination was well tolerated with no unexpected toxicity, and with preliminary evidence of clinical activity	Metastatic pancreatic cancer	NCT01130142 [129]
LDE‐225 (sonidegib) in combination with gemcitabine and nab‐paclitaxel. (MATRIX trial)	2015–2019	I, II	Hedgehog pathway	Completed Primary outcome measures: DLT Secondary outcome measures: Median survival, PFS The combination was well tolerated, and showed promising efficacy after prior treatment with FOLFIRINOX. LDE‐225 improved tumour diffusion (fMRI)	Pancreatic cancer	NCT02358161 [130]
Vismodegib in combination with gemcitabine (NEOPACHI‐001)	2012	I	Hedgehog pathway	Recruitment status: Unknown Outcome not published	Resectable PDAC	NCT01713218
LDE‐225 (sonidegib) in combination with fluorouracil, leucovorin, oxaliplatin, irinotecan	2011–2020	I	Hedgehog pathway	Completed Outcome not published	Untreated advanced pancreatic cancer	NCT01485744
GDC‐0449 (vismodegib) and erlotinib with or without gemcitabine	2009–2022	I	Hedgehog pathway	Ongoing (Active, not recruiting) GDC‐0449 and Erlotinib, were tolerated at a dose of 150 mg each, and were suitable for evaluation at phase II	Metastatic or non‐operable pancreas cancer	NCT00878163
GDC‐0449 (vismodegib) in addition to gemcitabine	2009–2013	I, II	Hedgehog pathway	Completed Primary OM: PFS Secondary OM: OS, ORR, Adverse events, Overall response rate Addition of vismodegib to gemcitabine did not improve the overall response rate, PFS, OFS	Metastatic pancreas cancer	NCT01064622 [28]
LDE‐225 (sonidegib) in combination with gemcitabine and nab‐paclitaxel	2011–2020	I, II	Hedgehog pathway	Study terminated (manufacturing of study drug ceased)	Borderline resectable pancreatic cancer	NCT01431794
***Other pathways***						
Am80 (tamibarotene) (MIKE‐1)	2021–2025	I, II	Meflin (Reprogram pCAFs to rCAFs)	Primary OM: DLT (phase I), RR(phase II) Secondary OM: AE, OS, PFS Ongoing (Recruiting)	Unresectable PDAC	NCT05064618 [37, 39, 51]
Gemcitabine + Nab‐paclitaxel to target stroma	2011–2013	II	Stroma (Density) and tumour vessels and metabolism.	Completed Primary Outcome: effect on: stroma density, tumour vessel formation, tumour metabolism on PET‐CT. Secondary Outcome: activity against PDAC Outcome not published	PDAC	NCT01442974
Pamrevlumab + gemcitabine + nab‐paclitaxel or pamrevlumab + FOLFIRINOX	2019–2023	III	CTGF	Ongoing (recruiting) Primary OM: OS, R0 and R1 resection achieved. Secondary OM: EFS, PFS Outcome not published	Locally advanced unresectable pancreatic cancer	NCT03941093
Losartan + FOLFORINOX + proton beam radiation	2013–2020	II	Angiotensin receptor (targeting reduces collagen and hyaluronan levels)	Active, not recruiting Primary OM: R0 resection proportion. Secondary OM: PFS, OS, toxicity, rate of downstaging, QoL Combination resulted in downstaging of locally advanced PDAC, with associated R0 rate of 61%	Locally advanced PDAC	NCT01821729 [131]
Losartan and nivolumab in combination with FOLFIRINOX and SBRT	2021–2025	II	Angiotensin receptor (targeting reduces collagen and hyaluronan levels)	Ongoing (recruiting) Primary OM: R0 resection proportion. Secondary OM: PFS, OS, Pathologic complete response, SAE Outcome not published	Localised pancreatic cancer	NCT03563248
Simtuzumab + gemcitabine	2011–2015	II	LOXL2	Completed Primary OM: PFS Secondary OM: OS, Objective response Addition of simtuzumab to gemcitabine did not improve clinical outcomes	Metastatic PDAC	NCT01472198 [132]
Pembrolizumab without or without defactinib	2019–2023	II	FAK PD‐L1 (Immunotherapy combination)	Ongoing (recruiting) Primary OM: Pathologic complete response Secondary OM: OS, DFS, drug related toxicities Outcome not published	Resectable PDAC	NCT03727880

Pertinent to mixed results from trials, studies delineating the impact of stromal targeting have often relied on preclinical mouse models. In many cases tissue‐specific Cre‐lox conditional targeting is used to delineate the importance of pathways in specific stromal cell types on tumour biology. While informative, it is important to recognise that drug interventions will in most cases target malignant and stromal compartments, which can dramatically alter the outcome. As an example, targeting FAK genetically in FSP‐positive CAFs resulted in metabolic switching of the malignant tumour, more aggressive growth, enhanced inflammatory chemokine signalling, and a switch in tumour metabolism [58]; FAK is a key regulator of myofibroblast function and this study concurs with other interventions suppressing myCAF function in PDAC tumours [25, 26, 29, 30]. In contrast, targeting FAK systemically with kinase inhibitors in KPC mice targets both tumour and stroma, resulting in reduced fibrosis and tumour growth, and an enhanced response to both chemo‐ and immunotherapy [22]. Interestingly, the suppressive effect on CAFs and fibrosis is primarily driven by inhibition of FAK in tumour cells, to limit paracrine activation of the stroma.

Interestingly, a number of therapeutic approaches that suppress CAF functions appear to induce EMT signatures in malignant cells, with enhanced invasion and/or metastasis [29, 30, 93]. Counterintuitively, suppressing the contractile and invasive capacity of CAFs can promote more aggressive invasive behaviour of cancer cells. In the context of pharmacological intervention, it is noteworthy that pathways driving migration and invasion are likely to be shared by migratory cancer cells and fibroblasts, so drugs targeting myofibroblast‐led invasion are also likely to impede tumour cell invasion. In a variety of mouse models, targeting Rho‐associated kinase (ROCK) has been reported to block activation of CAFs, induce matrix remodelling. and also impede cancer‐cell migration [138, 139, 140, 141, 142]; a dual impact on CAFs and cancer cells appears likely to contribute to the efficacy of these compounds. ROCK‐targeting compounds used in these studies, including Y27632 and fasudil, also target the Rho‐effector kinase PKN2, which also regulates migration and invasion of mesenchymal cancer cells and fibroblasts [30, 143, 144]. Many additional pathways involved in mesenchymal invasion are also likely to represent dual targets in both cancer and stroma, including Rho family members, integrins, FAK, and the mechanotransduction apparatus. As a broader lesson, genetically engineered mouse models that target specific compartments to understand the biological contribution of specific cell types to tumour biology, will not model the impact of targeting signalling cascade pharmacologically across all tumour compartments. This may lead to apparent contradictory results from genetic manipulation in transgenic models versus drug targeting with small molecules or antibodies, as well as in combinatorial approaches.

Chemotherapeutics or targeted therapies aimed at killing or suppressing cancer cell growth can also have a significant impact on the stroma. Erstad *et al* demonstrated that fibrosis associated with FOLIRINOX (oxaliplatin, 5‐FU, leucovorin, and irinotecan) and radiation therapy predicts better patient outcome in pancreatic cancer [145]. Although pretreatment controls are lacking in that study, FOLFIRINOX also reduced tumour size and enhanced fibrosis in two murine syngeneic orthotopic models. Related to immunotherapy, the PARP inhibitor olaparib, used in the treatment of BRCA mutant cancers has been shown to have a beneficial impact on T‐cell targeting by modulation of SDF1α (CXCL12) production by CAFs [146]. In a less fortuitous example, targeting of BRAF mutant melanoma with the BRAF kinase inhibitor PLX4720 also drives activation of a fibrotic stromal response, which can result in therapy resistance [147]. Here the paradoxical activation of the Raf–ERK pathway in CAFs by PLX4720 drives integrin‐FAK‐mediated matrix production to protect the malignant epithelium. These studies highlight the importance of taking a holistic view of the impact of therapy on tumour biology. While mouse models can be invaluable in understanding the mechanism of action, most clinical therapies will target the malignant epithelium, stromal cells, immune infiltrate, and the systemic immune system, which can all impact therapy response.

## Stromal roles for CAFs are context‐specific

Sc‐RNAseq of primary tumour biopsies has revealed the potential for therapeutic stratification based on detailed subtyping and stromal analysis [83], although this technology remains some distance from clinical application. A number of studies have classified PDAC into distinct subtypes, based largely on bulk transcriptomic data, with an impact on prognosis, therapy response, and tumour pathology [148, 149, 150]. In a landmark study, Moffitt *et al* [148] used a bioinformatic approach to virtually dissect tumours to identify distinct stromal signatures in PDAC from bulk RNAseq data. Importantly, this demonstrated that an activated ‘myofibroblast CAF‐like’ stromal signature was independently associated with poor outcome. Importantly, however, the prognostic power of the stromal signature was also PDAC subtype‐dependent, showing good prognostic power in classical‐subtype PDAC but no power in basal‐like PDAC [148]. It might be surmised that in basal‐like PDAC, malignant cells may intrinsically exhibit more invasive characteristics and therefore the impact of invasive activated stromal CAFs may be diminished. With regard to mutation status, gain‐of‐function TP53 mutation has been shown to drive the generation of specific prometastatic CAF populations, which can also protect cancer cells from therapy, at least in part through modulation of the matrisome [151]. Targeting the stroma to impact therapy response can thus be influenced by both mutational and the disease subtype context. Layered on to this, multiple disease subtypes coexist within individual tumours, in spatially resolved CAF‐regulated microenvironments [83, 84, 85]. Successful targeting of CAF function must be tailored to both tumour and stromal signatures if response rates in trials are to be improved. Preclinical studies, where disease genetics are uniformly controlled, demonstrate the promise of stromal targeting, but we cannot expect these to model the heterogeneity seen in advanced disease in patient populations.

The challenge is to identify which patients are likely to benefit from specific CAF/stromal targeted therapy, and in the context of which anticancer therapeutic strategies. Overlapping tumour‐promoting and tumour‐suppressing roles, coupled with a context dependence of stromal interventions, must be considered. In clinical trials, where heterogeneity, within the tumour, stroma, and the patient population, generates many variables, and the results have unsurprisingly been mixed, with no stromal therapies adopted in mainstream clinical practice. Furthermore, studying large cohorts of human cancer samples will enable better understanding of stromal heterogeneity. Currently, human primary CAF characterisation studies involve only a handful of patients, although the translational value is clear [35, 83]. Progress will be critically supported by meta‐analyses of existing trial data; in trials where response rates are poor, the focus now falls on identifying parameters linked to therapeutic responses. Identification of specific CAF subtypes and stromal signatures have been successful at identifying CAF signatures associated with immunotherapy response, and these can be brought to bear in the clinic [64, 65, 84]. Only this more informed evidence ‐ased approach will improve the appropriate recruitment and success of clinical trials and subsequent tailoring of clinical pathways. Currently, initiatives of personalised medicine for anticancer treatment such as MSKCC‐Impact [152], Precision‐Panc [153] (NCT04161417), FOCUS‐4 (NCT03770468), TRACERx, (NCT01888601) rely on genome or transcriptome analysis focusing on the tumour cell compartment. We envisage a future where the whole TME will be taken into account, whilst delivering anticancer treatment.

## Author contributions statement

SM and AJMC were responsible for the focus of the review and wrote the article. MHO was responsible for reviewing and summarising current and past clinical trials targeting CAFs in PDAC and other cancers, and critically edited the article. SMAJ was responsible for contributing to writing, and reviewed CAF heterogeneity in breast cancer. HMK was responsible for contributing to writing and critically editing the article to provide clinical context.
